# Introduction of Mobile Health Tools to Support Ebola Surveillance and Contact Tracing in Guinea

**DOI:** 10.9745/GHSP-D-15-00207

**Published:** 2015-12-15

**Authors:** Jilian A Sacks, Elizabeth Zehe, Cindil Redick, Alhoussaine Bah, Kai Cowger, Mamady Camara, Aboubacar Diallo, Abdel Nasser Iro Gigo, Ranu S Dhillon, Anne Liu

**Affiliations:** ^a^​ Earth Institute at Columbia University, New York, NY, USA; ^b^​ Millennium Promise, New York, NY, USA; ^c^​ Millennium Promise, Conakry, Guinea; ^d^​ Brigham and Women’s Hospital, Boston, MA, USA; ^e^​ National Ebola Coordination Unit, Conakry, Guinea

## Abstract

Challenges in data availability and quality have contributed to the longest and deadliest Ebola epidemic in history that began in December 2013. Accurate surveillance data, in particular, has been difficult to access, as it is often collected in remote communities. We describe the design, implementation, and challenges of implementing a smartphone-based contact tracing system that is linked to analytics and data visualization software as part of the Ebola response in Guinea. The system, built on the mobile application CommCare and business intelligence software Tableau, allows for real-time identification of contacts who have not been visited and strong accountability of contact tracers through timestamps and collection of GPS points with their surveillance data. Deployment of this system began in November 2014 in Conakry, Guinea, and was expanded to a total of 5 prefectures by April 2015. To date, the mobile system has not replaced the paper-based system in the 5 prefectures where the program is active. However, as of April 30, 2015, 210 contact tracers in the 5 prefectures were actively using the mobile system to collectively monitor 9,162 contacts. With proper training, some investment in technical hardware, and adequate managerial oversight, there is opportunity to improve access to surveillance data from difficult-to-reach communities in order to inform epidemic control strategies while strengthening health systems to reduce risk of future disease outbreaks.

## Abstract (precis)

An informatics system consisting of a mobile health application and business intelligence software was used for collecting and analyzing Ebola contact tracing data. This system offered potential to improve data access and quality to support evidence-based decision making for the Ebola response in Guinea. Implementation challenges included software limitations, technical literacy of users, coordination among partners, government capacity for data utilization, and data privacy concerns.

## BACKGROUND

The longest and deadliest Ebola epidemic in history has prompted a historic global response in Guinea, Liberia, and Sierra Leone, the 3 countries affected most by Ebola. The epidemic began in December 2013 and has spread to 7 countries, causing, as of May 2015, more than 25,000 infections and almost 11,000 deaths.^1^

Controlling the epidemic requires a series of interventions: (1) community engagement; (2) identification of contacts; (3) contact monitoring for symptoms; (4) rapid lab confirmation of cases; (5) isolation and treatment of new cases; and (6) safe and dignified burials. Each activity is intrinsically complex, yet all need to be synchronized to stop transmission and control the outbreak.^2^ This challenge is compounded by the dynamic nature of an epidemic that is constantly evolving with transmission shifting to different environments. In this setting, real-time data become indispensable for guiding strategy, coordinating multiple interconnected interventions, and troubleshooting breakdowns in execution.

Real-time data in emergency situations are indispensable for guiding strategy, coordinating interventions, and troubleshooting problems.

Lessons learned from the mobile health (“mHealth”) community demonstrate a possible opportunity to address some of these gaps. mHealth tools have been used in low-resource settings for a variety of applications, including strengthening access to data, managing health records, and improving the quality of heath interventions.^3^^-^^5^ There have been selected instances in which mHealth tools have also been applied in emergency responses and for crisis management,^6^^-^^8^ but unfortunately quick adoption during times of emergency remains limited.

We established a real-time information system for contact tracing activities in Guinea. This system comprises a smartphone application that supports contact tracer workflow and enables rapid submission of surveillance data, which is linked to a business analytics and visualization software. Dashboards built on this platform facilitate access to the data by government response managers based in both prefecture-level offices and within the National Ebola Coordination Unit (NECU). The system allows for: (1) improved quality of work through built-in algorithms for contact tracers to follow; (2) real-time identification of contacts who have not been visited, allowing same-day intervention; and (3) stronger accountability of contact tracers through timestamps and collection of GPS points with their surveillance data. In this article, we describe the opportunities and challenges of deploying an mHealth system in response to an epidemic based on our experiences in Guinea during the Ebola outbreak.

An mHealth application for contact tracing in Guinea was developed to facilitate rapid surveillance of and response to the Ebola epidemic.

## PROBLEM

Contact tracing is the linchpin of epidemic control. It involves identifying all people who were exposed to someone with confirmed infection (termed “contact identification”) and monitoring them for the incubation period (21 days in the instance of Ebola). The purpose of the monitoring period is to diagnose and isolate contacts immediately if they develop symptoms (termed “contact monitoring”).^9^ With efficient contact tracing, the window of time during which an infected symptomatic person can transmit the disease is minimized, allowing for rapid disruption of human-to-human transmission.

In the current Ebola epidemic, contact tracing has been uniquely challenging due to the sheer numbers of cases and, thus, contacts; at one point, there were almost 4,000 contacts being monitored in Guinea spread over half of the country’s 33 prefectures.^10^ In addition, communities in affected countries are highly mobile, routinely traveling between different parts of the country. Furthermore, the communities may be resistant to contact tracing teams due to fear and stigma.

Contact tracing efforts in Guinea are conducted by contact tracers who are recruited locally and trained to monitor contacts in their community. Each contact tracer is required to visit their assigned contacts on a daily basis for 21 days to assess for symptoms of illness while also serving as a community resource for Ebola-related information. Contact tracers are managed by “close supervisors” who coordinate their activities and monitor whether contact tracers visit their assigned contacts through random household spot checks.

**FIGURE 1.**
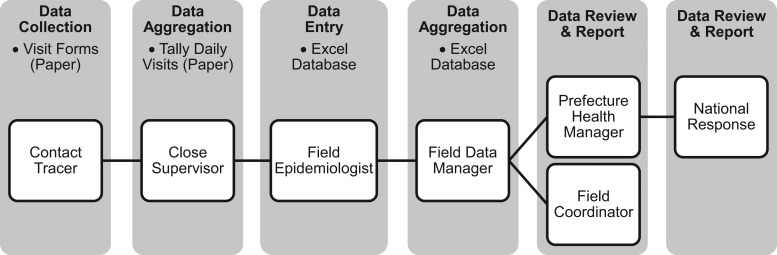
Flow of Information Using Paper-Based Contact Tracing System in Guinea

The current paper-based system proceeds as follows: The contact tracers use paper forms, based on internationally recognized templates,^9^ to record relevant information on the contacts they track daily. They then submit these forms to supervisors/field epidemiologists who collate them and enter the data into an Excel database. These data are aggregated at the prefecture level for review and submitted to national-level epidemiology teams for dissemination and analysis (Figure 1).

This paper-based system and the time-intensive effort required for entering, collating, and cleaning data pose several limitations (Table 1). First, there is a time lag of 2 to 3 days for data collected by contact tracers to be processed and available for managers to use. Second, it is difficult to identify points at which mistakes may have been introduced during any step of the process. Third, this process requires significant time and energy by response teams devoted solely to data entry, drawing resources away from analyzing and acting on the issues presented.

The paper-based system for contact tracing requires significant time and effort to enter, collate, and clean data, creating lag times in response.

**TABLE 1:** Limitations of Paper-Based Contact Tracing System

Limitation		Process Impact
Paper-based contact tracing system creates delays between data collection and consumption.	→	Impedes rapid response and decision making around contact tracing strategy.
Human error is common with data entry, as is misunderstanding of data and communication gaps.	→	Data become unreliable, out of date, and inconsistent.
Efforts for data cleaning, data entry, and data compilation are time-intensive.	→	Takes away from time and resources needed for data analysis and troubleshooting.

Collectively, these limitations undermine the ability of response managers to accurately understand which contacts are going unmonitored, to evaluate the performance of different contact tracers, to pinpoint where and why lapses are occurring, and to troubleshoot breakdowns. For example, if a contact tracer missed a contact visit or a contact could not be located, response managers may not find out until a few days later. Furthermore, given the urgent nature of the epidemic, many contact tracers were rapidly deployed and inconsistently trained, potentially leaving gaps in performance. Given how pivotal effective contact tracing is for reducing transmission, these delays and problems have significant consequences for controlling the epidemic whereby new chains of transmission may go unrecognized.

## SOLUTION

Implementation of the mobile contact tracing program involved 3 major stages:

1. Preparation
2. Deployment
3. Adaptation

Some steps within each stage ran concurrently. The length of time and key partners involved in each step are summarized in Table 2. The head of the Surveillance Pillar of the NECU led the decisions about the locations and staggered timeline for deployment—beginning in Conakry, the country’s capital, and then expanding by prefecture according to Ebola caseload. A team of 3 West African global health experts, 2 experienced with information technology and 1 in health systems in crisis settings, was deployed to Conakry by the project headquarters in New York to manage local deployment, provide continuous quality improvement, and oversee maintenance of the program.

**TABLE 2:** Mobile Contact Tracing Program Implementation Steps and Challenges

Stage	Step	Approximate Time	Key Partners	Prerequisites for Deployment	Key Challenges
Preparation^a^	1. Design of Application	1 week	Dimagi	• Vetted contact tracing protocols available from CDC, WHO, and GoG	• Availability of standard contact tracing protocol
	2. Development of Dashboards	4 weeks	Tableau Foundation, Tableau consultants, Dimagi	• Data requirements and desired indicators available from GoG and partners • Stable Internet and electricity available in country to access dashboards	• Interoperability of Dimagi’s data infrastructure and Tableau system
Deployment^a^	3. Procurement and Configuration of Equipment	2 weeks	Ericsson, UNFPA, Blue Zones	• Logistics protocols in place for accepting and processing equipment donations • Telecommunications network identified for highest coverage in different prefectures	• Manpower to configure phones • Logisticians to help clear donated equipment • Cash flow availability for voice and data plans
	4. Training of Trainers	2 days^b^	UNFPA, GoG	• Trainers pre-identified and hired by GoG	• Quality of some trainers
	5. Training of Contact Tracers and Supervisors	2–3 days^b^	UNFPA, GoG	• Contact tracers and supervisors pre-identified and hired by GoG • Contact tracers trained on contact tracing protocol • Supervisors trained on contact tracing protocol and supervision activities	• Accountability of supervisors to supporting CommCare troubleshooting needs • Gaps in training on basic contact tracing protocols • Technological literacy level of some contact tracers
	6. Deployment of Mobile Application	1 day	UNFPA, GoG	• Completion of contact tracer training • Distribution of smartphones, chargers, SIM cards, and other equipment to contact tracers • Contact tracer consent on proper-use protocols for smartphones and other equipment	• Quality, timely make-up trainings for contact tracers who were not able to attend initial training and were still expected to monitor contacts using smartphones • Abuse of phone and/or data by contact tracers for personal use
Adaptation	7. Data Validation	1 day^b^	UNFPA, GoG	• Contact tracing data from paper forms available and vetted	• Availability of up-to-date paper-based data • Quality of data from paper-based databases
	8. Modification of CommCare Application	1 week^b^	Dimagi	• Feedback on application content available from local partners and users based on use during pilot phase	• Conversion of nuanced field protocols to automated skip logic and prompts • Distribution of heavy multimedia files • Coordination of updates where application was already deployed
	9. Training and Deployment of Information Officers	1–4 weeks^b^ depending on applicants	GoG	• Interest and willingness from GoG to work with information officers within commune or prefecture-level offices • Internet access in government offices where information officers would be working • Space for information officers to work in government offices	• Availability of qualified applicants in rural areas • Management of expectations with GoG, with some information officers having to support directly on supervision • Some transportation gaps in rural areas where information officers had to support on direct supervision

Abbreviations: CDC, US Centers for Disease Control and Prevention; GoG, Government of Guinea; UNFPA, United Nations Population Fund; WHO, World Health Organization.

a Many of these steps occurred concurrently.

b Per prefecture.

### Stage 1: Preparation

#### Design of Application Software

We chose to use the Open Data Kit (ODK)-based software CommCare from Dimagi as the data collection tool for contact tracing activities. Although there were other mobile health options that were being considered for use in Guinea, there were several features of CommCare that guided our decision. CommCare is one of the few mobile health applications that allows longitudinal tracking of individual people over time—a feature critical for a surveillance system that required daily visits over a set period of time—and can also accommodate the reassignment of contacts and their history from one user to another if a contact tracer needs to be switched. Furthermore, with its algorithm-based decision support features, the software has been used to reinforce maternal and child health tracking in other projects managed by the implementation team since 2013. The implementation team’s familiarity with the software’s strengths and weaknesses was also a factor that contributed to the selection of the software, as it enabled rapid implementation and minimal software iteration. Key functions of CommCare that were leveraged for the program are summarized in the Box.

**BOX. Key Functions of CommCare According to Contact Tracing Goals**

**Goal 1: Reduce Time Delay Between Data Entry and Data Consumption**

- Data transmits to the server upon Internet access (Edge, 3G, Wifi). If a user is not in an area with network coverage, the data are saved to the phone and automatically sent upon entering an area with network coverage.
- All data collected in the system are available for export to CSV or Excel files. Additionally, the data are automatically linked to dashboards with predefined analytical charts.
- All users with the appropriate permissions and with Internet connection can access the data as soon as the data are synchronized to the server. Permissions can be set based on location and data management needs. In areas of low bandwidth, reports can be set up to be sent automatically via email.

**Goal 2: Strengthen Quality of Work of the Contact Tracers and Quality of Data**

- Preset contact tracing algorithms with built-in skip logic can guide a contact tracer through visits by prompting key questions based on previously inputted answers.
- Real-time data on performance (number of visits per day, GPS points, etc.) can inform supervisors for monitoring and supporting the contact tracers.
- Multimedia files including image, audio, and video can assist contact tracers in sensitization activities and can be used for refresher training as needed.

CommCare is one of the few mHealth applications that allows longitudinal tracking of individual people over time.

We created the contact tracing application using protocols that were publicly available from the US Centers for Disease Control and Prevention (CDC) and the World Health Organization (WHO),^9^^,^^11^ and then revised it in consultation with local United Nations (UN) agencies and government partners to reflect Guinea-specific procedures. The application consists of 3 forms that:

1. Register contact details upon first visit by the contact tracer
2. Follow-up with contacts daily for 21 days to monitor symptoms
3. Close the contact from the system once the 21-day period has been completed or for other reasons, e.g., the contact has been confirmed to have Ebola or has moved permanently to another area

This application structure thus enables users to follow contacts for the duration of the disease’s 21-day incubation period. Additionally, the application recommends action steps based on data entered in the form. For example, if a contact develops symptoms, the application reminds the contact tracer that the contact should be immediately referred for testing and treatment (if positive). A contact tracer would then follow-up with the family and/or a supervisor to confirm the test results. If confirmed for Ebola, the diagnosis would inform the closing of the contact from the system; otherwise, the contact would only be closed after completing the 21-day monitoring period upon returning home. Audio and visual content translated into local languages was also added to support contact tracers for Ebola sensitization activities. During the initial deployment of the application, publicly available audio and visual sensitization resources from WHO, Doctors Without Borders, and the CDC were included. The application was later updated with multimedia content tailored to the Guinean context, developed by headquarters in New York in consultation with the local team, over the course of 3 months. Lastly, a training video module was included to provide contact tracers with ongoing assistance if they needed refresher information on how to use the system.

#### Development of Dashboards

**FIGURE 2.**
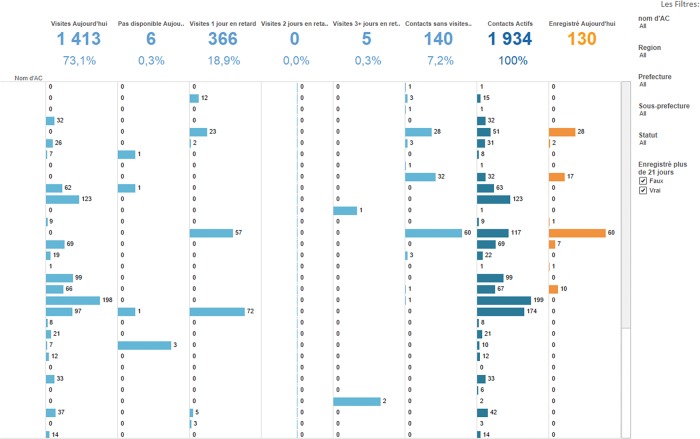
Contact Tracer Performance Dashboard Numbers at the top depict the numbers of contacts for the day that: (1) received a household visit from the contact tracer; (2) were visited by the contact tracer but were unavailable; (3) were last visited by the contact tracer 1 day ago; (4) were last visited by the contact tracer 2 days ago; (5) were last visited by the contact tracer 3 or more days ago; (6) have never received a monitoring visit since being registered in CommCare; (7) are active in the system and should receive a daily visit; (8) were newly registered in the system. The username of the contact tracer is displayed in the column on the far left but the names have been removed to maintain confidentiality. The filters on the right include: the name of the contact tracer; the region, prefecture, and sub-prefecture of the contact; the status of the contact (either active or not active); and whether the contact was registered more than 21 days ago. A user with appropriate permissions can then click on any bar in the graph to view the underlying specifics, i.e., the name of the contact, the date of the visit, whether they displayed any symptoms, etc.

To facilitate interpretation of the data collected in CommCare, the business intelligence software Tableau was integrated with the CommCare server. This software was recommended by Dimagi as a powerful analytic tool that could be integrated with CommCare. Once the key fields in the application were established, we began designing draft dashboards to visualize the data. Two sets of dashboards were developed by volunteer Tableau experts over the course of 1 month:

1. In one set, 3 dashboards depict aggregate indicators that are directly collected in each of the 3 forms, such as the relationship between the source case and the contact and symptoms presented by the contacts.
2. The other set of 2 dashboards presents information on the performance of the contact tracers, including the number of visits conducted by each contact tracer per day (Figure 2).

**FIGURE 3.**
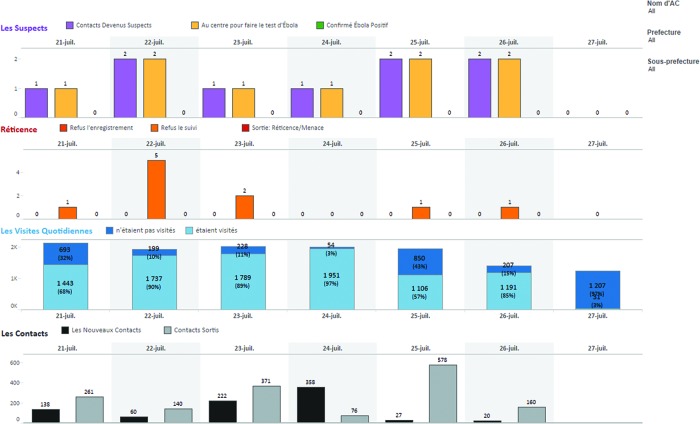
Weekly Key Performance Indicator Dashboard Four graphs are depicted, each showing data for the date specified, in this case, July 21– July 27, 2015. The top graph labeled “Les Suspects” shows the number of contacts who are calculated as suspected of having Ebola based on the display of symptoms, the number of contacts who have been transferred to a health center to be tested for Ebola, and the number of contacts who have been closed in the system because they were confirmed as having Ebola. The next graph labeled “Réticence” depicts instances of contact resistance during the initial registration visit and during the daily monitoring visit, as well as contacts who have been closed from the system and who will no longer be monitored because of resistance. The graph labeled “Les Visites Quotidiennes” depicts the number and proportion of contacts who are active and who have received their daily monitoring visit compared with those who were not visited for the day. The bottom graph titled “Les Contacts” shows the numbers of newly registered and closed contacts per day. The filters on the right allow the user to restrict the data to a specific contact tracer based on the username or to a specific prefecture or sub-prefecture. With proper access permissions, the user can click on any bar in the graphs to view the underlying specifics of the data, i.e., the username of the contact tracer and the name of the contact.

We also designed a Key Performance Indicator (KPI) dashboard with the Tableau experts, which displays a summary of data for the past 7 days (Figure 3). Filters can be set by users to selectively display data according to time period, e.g., the past week or geographic zone (such as a sub-prefecture of interest), among other variables.

The link between the CommCare and Tableau software made by Dimagi’s engineers and the Tableau experts allows contact tracing data to be updated on the dashboards automatically every hour. Users can view the visualizations by logging into the secure online Tableau server. The underlying specifics of the data, i.e., the personally identifiable information for each contact registered in the system, are also viewable in Tableau for authorized users and are linked to the aggregate dashboards. Thus, if users with the appropriate permissions notice that many instances of resistance were reported in the past week, they can navigate from the aggregate graph directly to the details page, where the resistant contacts will be listed and can be identified for follow-up. Access to the Tableau server was granted to all government health staff managing surveillance activities at the prefectural and regional levels, to members of the NECU, and to any workers from partner organizations and agencies as requested by the NECU.

Data analytic dashboards are updated automatically every hour, enabling surveillance managers to quickly identify trends and gaps.

The system’s features thus enable surveillance managers to directly interact with and use the data to quickly identify trends and gaps. This rapid data intake and ease of interaction allows for faster response. This is in contrast with the paper system’s outputs that include: (1) daily, aggregate, static visuals for the NECU, and (2) inconsistently updated Excel files that capture paper-form entries, which require additional manual analysis to identify trends.

### Stage 2: Deployment

#### Equipment Provision

Ericsson donated an initial 1,000 Sony Xperia E1 phones, and the UN Mission for Ebola Emergency Response (UNMEER) then donated an additional 580 Samsung Galaxy S3 Neo and Motorola ATRIX phones. Solektra International donated Sunking Pro2 solar chargers to facilitate phone charging, as well as 33 solar panels to enable regular access to power in the government offices where CommCare data are being used. The United Nations Population Fund (UNFPA) helped facilitate the purchase of SIM cards with a closed user group and 500 MB of data per month from Orange. Our implementation team also provided computers and USB modems to government staff as necessary, to ensure regular access to data.

#### Training of Trainers

Trainers were selected by the government and local UN agency partners from a pool of governmental health staff who supervise contact tracing activities. The first training of trainers (ToT) was conducted in Conakry by the local program staff in November 2014 and included approximately 17 trainers. The first half of the ToT included a didactic introduction to the phones and CommCare generally, while the second half of the ToT used blended learning techniques,^12^ incorporating multimedia, role-playing, and peer teaching to build expertise in using the CommCare application. Topics included contact registration, data input requirements for each form, GPS position collection, and form submission.

#### Training of Contact Tracers and Supervisors

Upon completion of the ToT, trainers were divided into pairs based on geographic area of supervision to conduct 2-day trainings with contact tracers and close supervisors. Because contact tracers had already received training on basic contact tracing activities, this training focused on the mobile health application. The training started with a brief review of the importance of contact tracing, safety, Ebola danger signs, transmission, and prevention, as well as tips for respectfully engaging with households. Trainers then provided an overview of the purpose and structure of the CommCare application, the types of data collected in CommCare, how to use the application, and how the data would be used. The second day of the training served as a practicum during which trial phones were distributed for practice sessions. Following initial training, periodic refreshers have been conducted as needed, especially with the objective of engaging supervisors in troubleshooting and monitoring data quality issues reported by local staff. Table 3 summarizes the training schedule and numbers trained.

**TABLE 3:** Mobile Contact Tracer Program Deployment in Guinea as of May 2015

Prefecture	Training Date(s)	Number of Contact Tracers Trained	Number of Supervisors Trained
Boffa	April 20–24, 2015	72	25
Conakry	Nov 11–17, 2014	105	13
	Jan 20, 2015	NA	9
	April 28–May 11, 2015	NA	38
Coyah	Nov 30–Dec 2, 2014	27	8
Dubréka	Dec 5–9, 2014	36	10
Forécariah	Mar 30–April 2, 2015	126	30
**Total**		**366**	**133**

#### Deployment of Mobile Application

At the end of the contact tracer training, participants were given unique usernames and passwords for CommCare, phones, SIM cards, and phone accessories, and they were asked to sign proper phone use agreements. Contact tracers who were already monitoring contacts were prompted to begin using CommCare to register their contacts and record information from daily contact monitoring visits in addition to the paper forms, which have remained the official reporting medium throughout the crisis. In certain prefectures, the training ended with the contact tracers registering all current contacts as a group. Contact tracers were also instructed through the application to obtain consent with each visit. To date, contact tracers in 5 prefectures—Boffa, Conakry, Coyah, Dubréka, and Forécariah—are using the CommCare system to monitor contacts of Ebola cases. As of May 2015, 210 contact tracers from the 5 prefectures were actively using CommCare to monitor more than 9,000 contacts collectively (Table 4).

**TABLE 4:** Use of the Mobile Contact Tracer System in Guinea as of May 2015

Prefecture	No. of Months Since Initial Deployment	Total No. of Active Contact Tracers^a^	Total No. of Contacts Monitored in CommCare
Boffa	0.5	5	77
Conakry	5.5	89	6,151
Coyah	5.0	23	750
Dubréka	4.5	25	619
Forécariah	1.0	68	1,565
**Total**		**210**	**9,162**

a Defined as having registered contacts using CommCare.

Once the application was in use, the prefecture-based surveillance teams and our implementation team members began using the Tableau dashboards to monitor contact tracer activities. Initially, these surveillance teams mostly used the dashboards that focused on contact tracer performance, which functioned as a quick mechanism to see which contacts had not received daily visits. As the numbers of contacts has diminished and familiarity with the system has grown, prefecture-based teams are increasingly using the system to identify trends, for example, to investigate areas where resistance is high or zones where re-exposure to Ebola is frequent.

### Stage 3: Adaptation

#### Data Validation

To test the accuracy of the CommCare system, validation exercises compared CommCare raw data with paper-based contact tracing databases from the government for a given week for each commune or prefecture. When contacts could not be matched by first name, last name, age, and village, we would confer with the contact tracers and supervisors to understand the reasons for discrepancies.

During the first round of data validation for the 5 communes of Conakry, there was 78% agreement between the 2 databases. In response, we made programmatic adjustments to reinforce supervision and improve communication with partner organizations. For our second round of validation in Conakry, we reached 86% agreement. In general, missing data were attributed to 4 main deficiencies:

1. Training shortcomings/misunderstanding of the software
2. Miscommunication with partners who manage contact tracers
3. Misuse of phones
4. Errors in the reference database from the paper system

To address the first 3 deficiency categories, we changed hardware and SIM card management protocols and conducted targeted refresher trainings. Challenges from the fourth category highlighted potential weaknesses in the original paper system in which data entry errors, including duplicates, created misconceptions about contact numbers.

#### Software Adaptation

**FIGURE 4.**
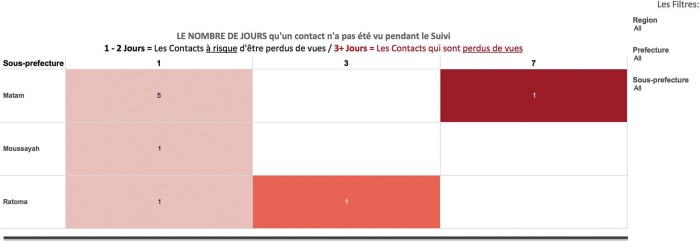
Contacts Lost to Follow-Up Dashboard This graph shows the number of days that have passed since contacts were available and visited by the contact tracer and were available at the household to be monitored for symptoms. (Note: Once a contact has not been seen for 3 or more days, they are considered lost to follow-up by surveillance management teams.) The column on the left indicates the sub-prefecture or commune where the contacts reside. The numbers in the colored boxes show the number of contacts who were unavailable for the daily contact tracing visit since 1 day ago (left column), 3 days ago (middle column), and 7 days ago (right column). The filters on the far right enable the user to restrict the data to contacts who reside in a specific region, prefecture, or sub-prefecture. The user can also click in an individual box to see the specific details for the contacts, i.e., the assigned contact tracer, name of the contact, source-case name, household head name, method of exposure, date the contact was registered, date the contact was last available to be monitored by the contact tracer, etc.

We refined the design of the application and the accompanying dashboards as the epidemic evolved, largely due to the lack of standardized contact tracing protocols and metrics agreed upon by all partners. Clearer standards from the outset could have accelerated the design process. Instead, the results of the validation exercises and feedback collected from the field were used to inform at least 3 software adaptations every 2 months for application content, logic, and prompts to better align with on-the-ground practices. For example, new options and alerts were added to the forms to reflect events in the community, such as a contact moving to a new area or being transferred to a health facility for testing. Rollout of these application updates was accompanied by refresher trainings by the local implementation team. We also iteratively designed the dashboards based on evolving end-user needs. For example, as frequent resistance from and movement of contacts continued to be a key challenge facing the Ebola response in Guinea, a new dashboard was developed to allow local surveillance teams to quickly identify contacts who were lost to follow-up (Figure 4).

#### Deployment of Information Officers

Centralized management of the mobile contact tracing system was not sufficient to ensure quality data collection by the contact tracers and routine data use by government staff and partners. In response to these capacity issues, information officers with experience in data analysis and community health programs were hired, trained, and deployed to local governmental health offices where Ebola surveillance activities are coordinated. Their initial training included an overview of the mobile contact tracing system and how to use the Tableau dashboards for monitoring data submissions by the contact tracers. The information officers’ responsibilities include basic troubleshooting of the mobile phones, daily review of data submissions, and communication of key trends to local response teams. They also train and support government office-based staff in using Tableau to manage the Ebola response.

Information officers were hired to ensure quality data collection and facilitate routine data use by government staff and partners.

## UNRESOLVED QUESTIONS AND LESSONS LEARNED

Implementation of technology during an emergency outbreak is not without its challenges. The current iteration of the program is the result of a series of trials and corrective measures put in place to address the most common obstacles, detailed below.

### Software and Training Limitations

While the CommCare software incorporates data submission and workflow features meant to be used in low-resource health contexts, it was not designed explicitly for emergency use. Additionally, the structure of the software created limitations in capturing nuances of complex contact tracing protocols. Furthermore, messy data from the field that required additional editing in the backend of CommCare was not easily accommodated in Tableau—which is more commonly used for commercial activities in developed settings. Tableau experts and Dimagi’s engineers performed considerable troubleshooting remotely to refine and improve the connection between the 2 software programs. Finally, time limitations were also a challenge as the dashboards were built by Tableau experts who were volunteer consultants. Commissioning full-time Tableau consultants could have helped to shorten the time required to build the dashboards; however, they may not have had as much experience as the volunteer experts suggested by Tableau Foundation.

**Figure:**
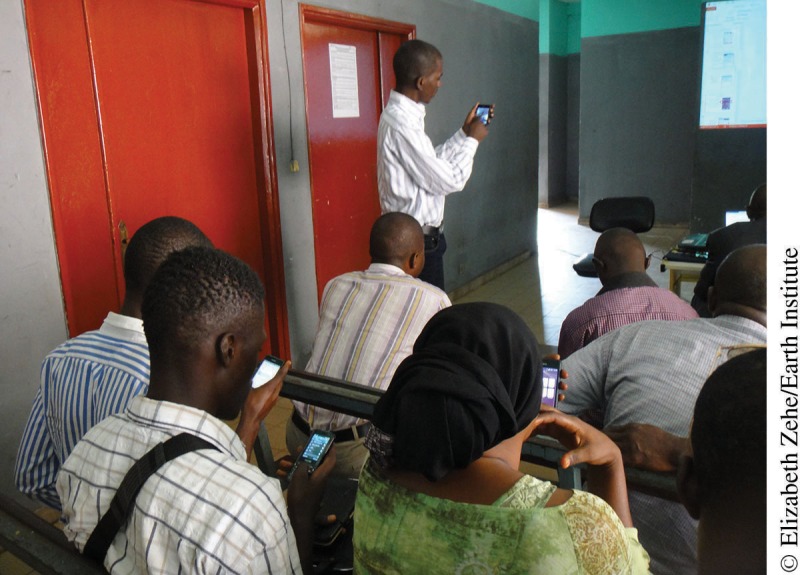
Contact tracers in Conakry, Guinea, attend a refresher training on the CommCare software.

### ICT Challenges and Hardware Management Policy

The availability of local staff to support phone configuration and hardware management was limited. Adjusting phone settings for language, date, and time and addition of application locking software and widgets for battery saving required between 10–15 minutes per phone. During the expansion phase, recruitment of local tech-savvy youth volunteers helped to accelerate the configuration of hundreds of phones.

After equipment was distributed, there were also limitations in implementing management policies for lost phones, data plan abuse, and phone storage. Because the contact tracers are co-managed by multiple entities including UN agencies, NGOs, and the government, coordination was necessary to create consistent policies that were agreed to and adopted by all local managers.

Finally, although the CommCare software could be used offline for data collection, access to the mobile network necessary for data submission was limited in the border regions of Forécariah prefecture, which affected 21 contact tracers. In these areas, we employed workarounds such as having the contact tracer submit data every few days when in locations with better network coverage or having the information officers directly request the data from the contact tracer via phone to enter themselves when necessary. An additional option, which has been used in previous projects but not in Guinea, is the manual transfer of data from the phone to an SD card, which could then be inserted to another phone for submission in areas with better network coverage.

### Use of Smartphones

The quality of data submissions by contact tracers was an issue at the outset. There were occasional instances where users—often older generation—were unfamiliar with smartphones and therefore could not use the software effectively. For example, some contact tracers submitted data under test user accounts that were used for training, or they made spelling errors. To address this, older-generation contact tracers would sometimes be paired with younger, more tech-savvy contact tracers or would receive more one-on-one support from implementation team members.

In some cases, contact tracers used paper forms to first record information at the household, which was then entered into the phones after the visit. Although we did not systematically collect data on the frequency of this practice, comments from the field indicated that this would occur when contact tracers were concerned that the introduction of foreign technology could affect their trust with community members, who may have found the act of data collection, especially through unfamiliar equipment, strange or threatening.

There were also a few cases of contact tracers misusing data plans for personal purposes. Eventually, a solution was developed in which a fund for personal phone use was added, and if contact tracers exhausted their monthly data allotment, additional data would be supplied using their personal accounts to ensure continued CommCare data submission. A monthly auto-recharge system was also set up to ensure uninterrupted submission of data.

### Partnerships, Funding, and Coordination

Coordination among partners was consistently a challenge that created difficulty in the identification of both contacts and contact tracers, access to paper-based data for validation, release of funds, integration of mobile contact tracing data with other Ebola response data, and cooperation with local supervisors who were managed by other organizations. International partners, including NGOs and UN agencies, expressed concern about the introduction of mobile phone technology for Ebola response purposes, citing poor literacy or possible network gaps as challenges. These apprehensions created significant delay in implementation and expansion despite having support and directive from the government lead of the Surveillance Pillar of the NECU. Validation exercises were conducted to address issues of data quality; however, presentations with partners were often delayed or canceled. Furthermore, the lack of clear leadership and coordination among partners, as well as the monthly rotation of leadership within key response organizations, created miscommunication and inconsistent messaging. To facilitate collaboration with other partners, our approach has been advocacy and transparency, offering to demonstrate the system and granting aggregate-level access to all agencies as requested by the government. Nevertheless, to date, the mobile system has not replaced the paper-based system in the 5 prefectures where the program is active, and many contact tracers still use both systems.

**Figure:**
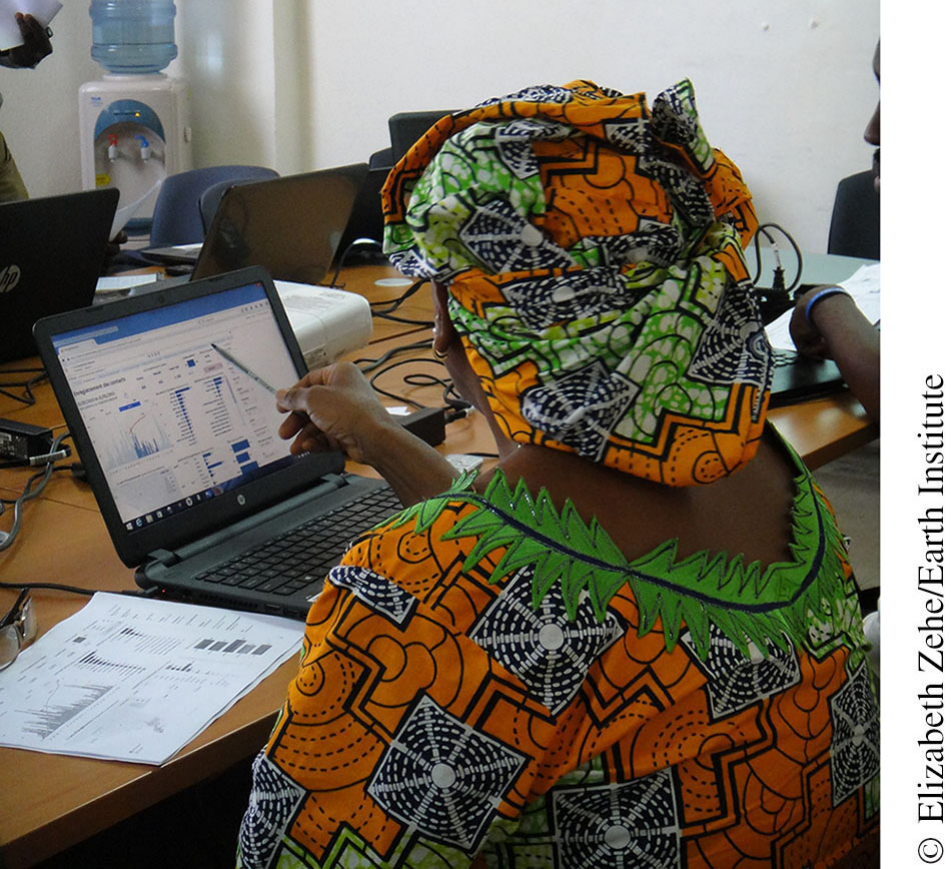
A government health manager in Conakry, Guinea, uses the Tableau dashboards to review new contacts registered in her commune.

### Data Utilization

While government staff members were especially enthusiastic about the wealth of data that could be used for supervision of contact tracers, actual use of the data was limited. Local government managers often had many competing interests and responsibilities, and thus relied instead on supervisors to report issues to them rather than exploring the data directly. While the corrective action of hiring information officers to be paired with government staff helped to increase the use of performance data, utilization of CommCare data to inform transmission trends and contact risk patterns has still been limited. It is important to recognize that the contact monitoring data being collected by this program are only one aspect of the response. Because each aspect of the response and the associated data are managed by different partners (i.e., call center, contact identification, contact monitoring, treatment centers, laboratories, safe burial teams), it is difficult to interpret broader trends from the contact monitoring data alone.

### Data Privacy and Ownership

As the data collected by CommCare contains protected health information as well as personally identifiable information, there were considerable concerns around data confidentiality. Although the Dimagi cloud server is compliant with the Health Insurance Portability and Accountability Act (HIPAA) and care was taken to ensure that data were shared in aggregate form, the national laws in Guinea around data privacy are still unclear. For this reason, datasets shared with partners have been mostly limited to aggregate indicators while government staff members have access to contact-specific details. Level of access has been determined by members of the NECU and facilitated by the implementing organization, which currently holds the end user license agreement with Dimagi per the government’s preference for ease of implementation. Formal transfer of data ownership to the government is in process and undergoing review by the NECU. There were also concerns from the government about data not being housed on a machine in country; for this reason a secondary local server was set up with a partner organization.

## CONCLUSION

Use of a smartphone-based mHealth application to assist with Ebola contact tracing activities has demonstrated the potential to improve access to surveillance data for informing response strategy. There is also opportunity to extend this type of methodology to other pillars of the Ebola response to improve rapid access to data and integration of key indicators in the response chain. While there are inherent challenges in introducing technology in complex emergencies, the benefits of innovation to disrupt the status quo may be integral to controlling long-standing epidemics. This being said, careful consideration must be given to whether it is feasible and beneficial to implement new technology during an ongoing outbreak. When the decision is made to implement technology, it is critical to accompany the deployment with close managerial oversight to quickly correct data inconsistencies and to address challenges.

A formal government digital health information system currently does not exist in Guinea to allow for rapid health data collection and analysis, and thus the mobile contact tracing program cannot directly be absorbed into another system. However, the government and UN agencies in Guinea have expressed interest in using digital technology for longer-term surveillance, especially if embedded in a strong primary health care system. Certainly, the operational costs needed to bring a mobile health program to national scale would be substantial and have to be formally considered. Many of the resources and time required to set up this system were donated by partner agencies for Ebola-specific work. This initial investment in human resources, in-country technical expertise, and hardware for the program can certainly be leveraged and expanded to strengthen health systems.

With proper technical capacity and oversight, mobile technology has the potential to expand access to critical data from remote communities needed to control an outbreak. As we have learned from this Ebola epidemic, it is essential to acknowledge and plan for stronger information systems that can detect and report health trends and aberrations. Accurate and readily accessible data can help health surveillance officers quickly identify and contain outbreaks. Furthermore, a strong information system can not only serve to prevent emerging epidemics but also be adapted and leveraged for activities such as contact tracing in the event of a disease outbreak. Thus, investments in strong community-based information systems should be considered as integral for strengthening health systems and emergency preparedness to prevent future epidemics as tragic as the 2014–2015 West Africa Ebola outbreak.

## Notes

**Peer Reviewed**
